# Camptothecin Induces PD-L1 and Immunomodulatory Cytokines in Colon Cancer Cells

**DOI:** 10.3390/medicines6020051

**Published:** 2019-04-24

**Authors:** Deepa Bedi, Henry J. Henderson, Upender Manne, Temesgen Samuel

**Affiliations:** 1 College of Veterinary Medicine, Tuskegee University, Tuskegee, AL 36088, USA; dbedi@tuskegee.edu (D.B.); hhenderson0533@tuskegee.edu (H.J.H.); 2 Department of Pathology, University of Alabama at Birmingham, Birmingham, AL 35294, USA; upendermanne@uabmc.edu

**Keywords:** camptothecin, colon cancer, cytokine, PD-L1, immunotherapy, topoisomerase inhibition

## Abstract

**Background:** Immunotherapy has changed the options for the treatment of various cancer types, but not colon cancer. Current checkpoint blockade approaches are ineffective in a large proportion of colon cancer cases, necessitating studies to elucidate its mechanisms and to identify new targets and strategies against it. **Methods:** Here, we examined Programmed Death-Ligand 1(PD-L1), cytokine and receptor responses of colon cancer cells exposed to camptothecin (CPT), a clinically used topoisomerase inhibitor. Colon cancer cells were treated with CPT at concentrations of up to 10 µM, and the expressions of PD-L1 and immunoregulatory cytokine genes and receptors were analyzed. **Results:** PD-L1, a current immunotherapy target for various cancers, was shown to be upregulated in colon cancer cells independent of the cellular p53 status. In metastasis-derived SW620 cells, CPT most extensively upregulated cytokines with T-cell attraction or growth factor functions. Of those modulated genes, SPP1, IL1RN, IL1A, TNFSF13B, OSM, and CSF3 had the most clinical relevance, as their high expression was associated with poor cancer patient overall survival. **Conclusions:** These findings highlight the need to examine, in preclinical and clinical situations, the potential benefits of combining topoisomerase inhibitors with immune-checkpoint inhibitors.

## 1. Introduction

Immunotherapy with checkpoint inhibitors is currently a promising approach for the control of cancer, with reports of complete and durable responses in multiple types of this disease. Although it holds promise for mismatch repair (MMR)-deficient and microsatellite-instable (MSI) subtypes of colorectal cancer CRC, those that are microsatellite-stable (MSS), which constitute the majority of CRCs, remain largely nonresponsive [1]. This is due, in part, to the lack of sufficiently distinct neo-antigens in MSS tumors, but other factors remain unexplored. 

In cancer cells, chemotherapeutic drugs may upregulate the expression of the PD/PD-L1 checkpoint pathway [2,3,4], but the mechanisms through which drugs increase the expression of these immunoregulatory proteins remain unknown. Immunogenic cell death has been suggested as a contributing mechanism to the benefits of combining radiation with immunotherapy [5]. Nevertheless, the upregulation of these regulatory molecules could be a mechanism through which cancer cells overcome a cytotoxic immune response [6,7]. Moreover, the increased expression of PD/PD-L1 has been linked to increased resistance of the expressing cells to chemotherapy [8,9,10]. Further, in some cases, the basal levels of these immunoregulatory proteins may not be sufficiently high to implement immunotherapy as a primary mode of therapy. 

Clinical associations between the abundance of immune cells or cytokines and cancer progression are not consistent. An abundance of immune cells is linked to both positive and negative outcomes [11,12,13,14,15], demonstrating that the mechanisms through which tumor-infiltrating immune cells are regulated are complex and incompletely understood. 

All cell types, including cancer cells, can produce cytokines in response to environmental cues. Our goal was to determine the nature of immunoregulatory molecular response in colon cancer cells exposed to CPT, a topoisomerase inhibitor used as a chemotherapeutic drug, and to explore the relevance of the induced proteins.

## 2. Materials and Methods 

### 2.1. Cells, Cell Culture, and Treatments 

SW620, HCT116, and RKO colon cancer cells were obtained from the American Type Cell Culture (ATCC) collection. Aliquots of subcultures from the original vials were stored in liquid nitrogen and retrieved for experiments. No further validations were done. HCT116 and SW620 cells were cultured in McCoy’s medium (Sigma-Aldrich or ATCC), and RKO cells were cultured in Dulbecco’s Modified Eagle’s Medium (DMEM). Both media were supplemented with 10% fetal bovine serum (FBS) and 10 μg/mL ciprofloxacin. Cells were incubated in a humidified incubator under 5% CO_2_ at 37 °C. Dimethylsulfoxide (DMSO, used as solvent for drugs) and ciprofloxacin were purchased from Sigma-Aldrich (St. Louis, MO, USA). 3-(2-bromoethyl)indole (BEI-9) was purchased from Sigma-Aldrich and dissolved in DMSO. Drugs used for the treatment of cells were obtained from Sigma-Aldrich (5-fluorouracil (5-FU), oxaliplatin, tumor necrosis factor alpha (TNFα), and troglitazole) or LC laboratories (erlotinib, phleomycin, and camptothecin (CPT)).

### 2.2. Cytokine Gene Expression and Analysis

Total RNA was extracted from cells using RNeasy extraction kits (Qiagen, Valencia, CA, USA). QuantiTect cDNA synthesis kits (Qiagen) were used to reverse-transcribe 1 μg of RNA in a final volume of 20 μL. RNA and cDNA were stored at −80 °C until used. The Human Cytokines & Chemokines RT^2^ Profiler PCR Array (Qiagen, catalog # PAHS-150ZA) was used to determine the expression of cytokine/chemokine genes modulated by treatment. For each sample, cDNA transcribed from 1 μg total RNA was distributed over 84 wells of a 96-well PCR array plate. Each of these wells contained primers for one cytokine/chemokine gene. The remaining 12 wells contained assay and reaction controls. Levels of gene expression were determined by the ΔΔCT method using the tools provided by the manufacturer (Qiagen). Altered expressions were determined if a ≥2-fold change was registered by treatment with either 1 µM or 5 µM CPT. A separate assay with the Human T-Cell and B-Cell Activation PCR Array (Qiagen PAHS-053Z) was run similarly, but treatment of cells was only with 1 µM CPT.

### 2.3. Gene Functional Annotation 

Since all the genes analyzed belonged to the same functional category (cytokines/chemokines), individual gene functions were collated from the GeneCards gene search database (www.genecards.org). Additional references on functions were cited for cytokine with major fold changes if specific experimental evidence was shown in the literature.

### 2.4. Bioinformatics Database Search and Analysis 

UALCAN [16] and University of California Santa Cruz (UCSC) Xena (https://xenabrowser.net) were used to interrogate gene expression and survival analyses. The colon adenocarcinoma (COAD) data set from TCGA and the PANCAN (pan cancer) dataset, with a duplicate entry removal tool (Xena), were used for the interrogations. Significance values were automatically generated by the in-built analysis tools. 

### 2.5. Immunoblotting

For immunoblotting experiments, cells were treated in 6-cm culture dishes and lysed in RIPA buffer, to which a cocktail of protease and phosphatase inhibitors was added. Samples containing equivalent protein concentrations were resolved by SDS-PAGE and analyzed by immunoblotting. X-ray films were used for the detection of chemiluminescent bands, except when the signals were strong, in which case a C-DiGiT Scanner (LI-COR^®^, Lincoln, NE, USA) or AI680 digital imager (GE Lifesciences, Pittsburg, PA, USA) was used to scan the signals. Primary antibodies, at 1:1000 dilutions, used to target PD-L1 (catalog #13684, rabbit mAb) and p53 (catalog #9282, rabbit), were obtained from Cell Signaling Technologies (Danvers, MA, USA). Rabbit anti-GAPDH antibody (catalog # TA802524, used at 1:2000 dilution) was from OriGene Technologies^®^ (Rockville, MD, USA). Peroxidase-conjugated anti-rabbit and anti-mouse IgG secondary antibodies were from Millipore (Temecula, CA, USA) and used at 1:3000 dilutions. 

### 2.6. PD-L1 Enzyme Linked Immunosorbent Assay (ELISA)

PD-L1 ELISA was performed using Human/Cynomolgus Monkey B7-H1/PD-L1 assay kits (cat # DB7H10) from R&D Systems (Minneapolis, MN, USA). The cell lysis buffer for this assay consisted of 2% NP-40, 40 mM Tris, 274 mM NaCl, 20% glycerol, 4 mM EDTA, and protease inhibitor cocktail (Sigma-Aldrich, St. Louis, MO, USA). Cell lysates were diluted in lysis buffer, and 40 µg of total protein was loaded per well. Optical density readings were set for dual measurement at 450 nm and 570 nm wavelengths. Readings at 570 nm were subtracted from those at 405 nm to obtain optical densities for data analysis.

### 2.7. Flow Cytometry for Surface Expression

SW620 cells were grown in 6-well plates. At 70–80% confluency, they were treated with CPT (1 µM) for 24 h. Cells were trypsinized and brought to a concentration of 10^5^ cells per 100 µL of blocking buffer (PBS containing 1% BSA and 1% triton-X100), followed by incubation with mouse anti–PD-L1 antibody (ab213524) (1:100) (Abcam, Cambridge, MA, USA) for 1 h at room temperature (RT). Cells were washed three times in washing buffer (PBS–1% BSA, 1% Triton-X-100, and 0.1% Tween-20) and then incubated with a secondary goat anti-mouse antibody conjugated with Alexa Fluor 488 (Life Technologies, Carlsbad, CA, USA) for 45 min at RT. Following three washes, cells were suspended in 100 µL of PBS and evaluated by flow cytometry (BD FACSCalibur, Becton Dickinson Biosciences, San Jose, CA, USA).

## 3. Results

### 3.1. CPT Potently Upregulates PD-L1 Compared to Other Drugs

**Figure 1:**
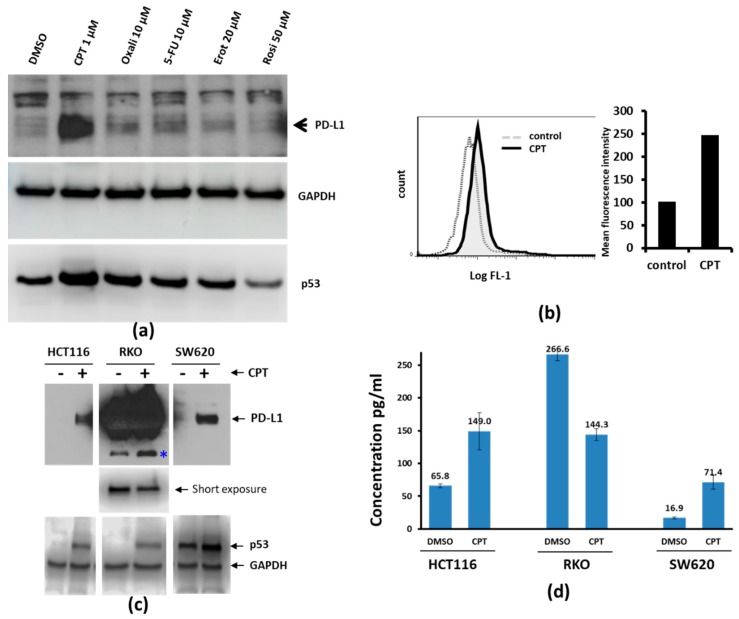
Camptothecin (CPT) treatment increases the expression of PD-L1. (**a**) SW620 colon cancer cells were treated with either vehicle or the indicated drugs for 24 h. Cell lysates were probed for the expression of Programmed Death-Ligand 1 (PD-L1) and p53. GAPDH was probed to compare the total protein loaded on the gel. Among the compounds tested under these experimental conditions, CPT was the best inducer of both PD-L1 and p53. (**b**) PD-L1 detection by flow cytometry. SW620 cells were treated with 1 µM CPT for 24 h, and the expression of PD-L1 was analyzed by flow cytometry. The solid black line in the left panel indicates the right shift in the fluorescence (FL1) peak, indicating increased expression of PD-L1 compared to control (dashed line). Quantification of the mean relative intensity for PD-L1 in control or CPT-treated SW620 cells is shown in a bar graph (right panel). Numbers below each panel indicate the relative integrated density of the protein band in that lane. (**c**) The three colorectal cancer (CRC) cell lines were treated with 1 µM CPT for 24 h, and cell lysates were examined for PD-L1 expression. The upper panel shows immunoblots for PD-L1. Due to the high levels of PD-L1 in RKO cells (middle panel), the protein bands were over-exposed. The middle panel shows a brief re-exposure of the PD-L1 immunoblot to show the levels of PD-L1. The lower panel shows immunoblot bands for p53 to indicate drug response and GAPDH for a loading control. * a possible cleavage product of PD-L1 in RKO cells. (**d**) ELISA assay for the determination of PD-L1 response in colon cancer cells. Cell lysates from DMSO- or CPT-treated cells were analyzed for PD-L1 levels by ELISA. HCT116 and SW620 lysates were loaded at 40 µg total protein per well, whereas 4 µg total protein was loaded per well for RKO cell lysates. The Y-axis and numbers above the columns of the bar graph indicate concentrations in pg/mL. Lysates were assayed in duplicate. Error bars show the standard variation from the mean.

We investigated whether drugs clinically used for CRC would upregulate PD-L1 expression. To address this, we exposed SW620 cells for 24 h to CPT, oxaliplatin, 5-FU, erlotinib (Epidermal Growth Factor Receptor (EGFR) inhibitor), or rosiglitazone (peroxisome proliferator-activated receptor (PPAR) inhibitor, control), and examined the expression of PD-L1 by immunoblotting. Among the compounds tested, only CPT upregulated PD-L1 (Figure 1a). We confirmed this by flow cytometry analysis for the cell surface expression of PD-L1, as shown by the shift in fluorescence intensity corresponding to the increased surface expression of the protein (Figure 1b). Although the relative protein upregulation seen by immunoblotting was very prominent compared to the surface detection by flow cytometry, it is possible that not all of the upregulated PD-L1 is anchored on the cell surface.

Additionally, two other CRC cell lines (HCT116 and RKO) were treated with CPT and the expression of PD-L1 was assessed. After CPT treatment, both HCT116 cells and SW620 cells showed upregulated PD-L1 protein (Figure 1c), with the protein prominently detectable after treatment. The levels of PD-L1 in the RKO cell line were high even without treatment. However, the level of PD-L1 in CPT-treated RKO cells appeared to be lower than the baseline level, and a protein band which appeared to be a breakdown product of PD-L1 (Figure 1c, *) increased, suggesting that the decrease of the major protein band is a posttranslational event. PD-L1 ELISA using cell lysates from control or CPT-treated SW620, HCT116, and RKO cells also showed similar results (Figure 1d).

### 3.2. Concentration-Dependent, NF-kB Activation-Independent Upregulation of PD-L1

**Figure 2:**
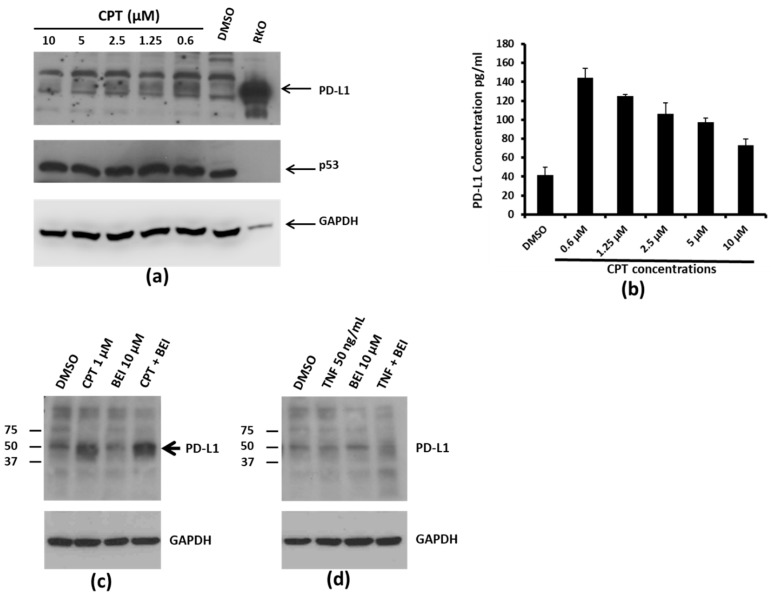
PD-L1 expression in response to CPT treatment. (**a**) SW620 cells were treated with the indicated concentrations of CPT for 24 h. The levels of PD-L1 in the cell lysates were evaluated by immunoblotting. p53 and GAPDH levels were also detected in the same lysates for drug response and for loading control, respectively. The RKO lysate was loaded on the same gel as a marker for detection of PD-L1, since RKO cells expressed the highest amounts of the protein per unit volume of lysate. Numbers below each panel indicate the relative integrated density of the protein band in that lane. (**b**) Cell lysates from SW620 cells treated with DMSO or increasing concentrations of CPT were prepared for ELISA. For this assay, 75 µg of total protein was loaded per well. The Y-axis of the bar graph indicates concentrations in pg/mL. (**c**) SW620 cells were treated for 24 h with DMSO or CPT, or with CPT followed by 3-(2-bromoethyl)indole (BEI, an Nuclear Factor kappa-light-chain-enhancer of activated B cells (NF-kB) inhibitory compound) for another 24 h. The expression of PDL-1 was detected by immunoblotting using an anti-PD-L1 antibody. (**d**) SW620 cells were treated with DMSO, tumor necrosis factor alpha (TNFα), or TNFα and BEI. PD-L1 expression was detected by immunoblotting. Neither TNFα nor cotreatment with BEI upregulated PD-L1 expression.

Interestingly, dose–response relationship experiments showed that PD-L1 levels did not increase with increasing concentrations of CPT. Instead, PD-L1 levels were highest at the lowest concentration of CPT used (0.6 µM), indicating an inverse relationship (Figure 2a,b). We have previously shown that the treatment of colon cancer cells with CPT may trigger Nuclear Factor kappa-light-chain-enhancer of activated B cells (NF-kB) signaling, which is inhibited by the indole compound, BEI-9 [17,18]. Therefore, to evaluate if TNF signaling or the blockage of NF-kB modulates the PD-L1 expression, the cells were exposed to CPT, BEI-9, TNFα, or the combination of CPT or TNF with BEI-9. As a result, only CPT upregulated the expression of PD-L1, and treatment with the NF-kB signaling blocker BEI-9 alone or in combination with CPT did not alter the upregulation of PD-L1 (Figure 2c,d). This suggested that the upregulation of PD-L1 by CPT may not be dependent on the NF-kB activating effect of CPT. Moreover, TNFα, a classical NF-kB inducer, did not alter the level of PD-L1.

### 3.3. Cytokine Genes Induced by CPT Treatment

Colon cancer immunotherapy based on PD—PD-L1 checkpoint regulators did not show desirable clinical efficacy. The mechanisms for this are not well understood. Hence, knowledge of the responses of colon cancer cells to conventional therapy may reveal targets suitable for combination therapy. To determine the nature of cytokine response by colon cancer cells, metastasis-derived SW620 cells were treated with vehicle (DMSO) or with 1 µM or 5 µM of CPT for 24 h and then analyzed by real-time PCR for the expression of 84 cytokines. Comparative analysis of the treatments revealed the differential expression of cytokine genes among the three groups (Table 1 and Supplementary Table S1). An overall comparison of the differential expression showed increases in expression for IL-12B (Interleukin 12B), LTA (Lymphotoxin Alpha), TNF (Tumor Necrosis Factor), IL-17F (Interleukin 17F), SPP1 (Secreted Phospho-Protein 1), and IL-24 (Interleukin 24) as the top-most upregulated (> 30-fold) cytokine genes after treatment. Additionally, IL-22 (Interleukin 22), CCL-21 (C-C Motif Chemokine Ligand 21), OSM (Oncostatin-M), IL-5 (Interleukin 5), and IL-16 (Interlukin 16) also showed a ≥4.5-fold increase in cells treated with 5 µM CPT. The levels of expressions of XCL1 (X-C Motif Chemokine Ligand 1), C5 (complement C5), IL-27 (Interleukin 27), and VEGFA (Vascular Endothelial Growth Factor A) were lower in CPT-treated cells in comparison to the control. Only FASL (Fas Ligand) and HPRT1 (Hypoxanthine Phosphoribosyltransferase 1) decreased by > 2-fold after treatment with 5 µM CPT (Supplementary Table S1). 

To understand the implication of the expressions of these cytokines, the functional annotations of those cytokines most modulated by exposure to CPT were examined. Annotated functions of the genes with a >2-fold change in expression were obtained from the GeneCards database. Twelve of the 22 cytokines (SPP1, IL-12B, CCL22, CXCL10, TNF, LTA, IL-17F, CCL17, CCL5, IL-22, IL-16, and CCL21) have functions directly related to the activation, recruitment, or migration of T-cells, or the promotion of inflammation. Of the remaining 10, six (CSF3, CCL2, IL-13, IL-5, IL-1A, and TNFSF13B (TNF superfamily 13B)) have functions targeting macrophages, granulocytes, or B-cells. 

The expressions of six genes (XCL1, C5, IL-27, VEGFA, and FASLG) were reduced in SW620 cells treated with CPT (Supplementary Table S1). Among these, XCL1 has a T-cell attractant function, and FASLG induces apoptosis in immune or other cells expressing the FAS receptor. IL-27 potentiates the TH-1 responses, appears to be anti-inflammatory, and antagonizes IL-17 responses. The roles of VEGFA and C5 in modulating leukocyte functions are less pronounced. The Human Cytokines & Chemokines PCR Array was utilized first to detect cytokine expressions which did not include PD-L1 among the tested genes. Therefore, a separate experiment was conducted with Human T-Cell and B-Cell Activation PCR Array (Qiagen PAHS-053Z). Consistent with our protein data above, PD-L1 (CD274) was upregulated 3.9 fold. IL-12B and CXCL8, present in both arrays, were also upregulated after 1 µM CPT treatment.

### 3.4. Clinical Relevance of CPT-Induced Cytokines

**Figure 3:**
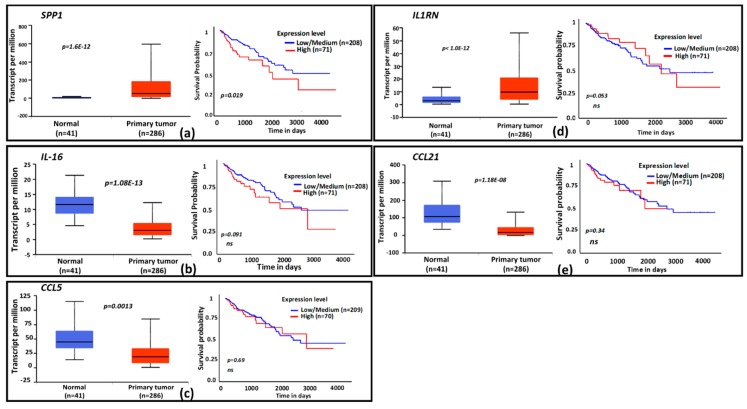
Expression profiles and functional categories of CPT-responsive genes and survival prognosis based on gene expression. Cytokine genes identified as CPT-responsive were probed for clinical significance in The Cancer Genome Atlas (TCGA) database. Graphs for the 5 genes with significant expression and/or survival probability in the database are shown. (**a–e**), The TCGA colon adenocarcinoma (COAD) database was probed for gene expression and patient survival profiles by using the UALCAN portal. Box plots indicate relative gene expressions between normal tissues and primary tumors; line graphs are Kaplan–Meier patient survival curves for relatively low (blue) or high (red) gene expressions. Genes: (**a**) = *SPP1*, (**b**) = *IL-16*, (**c**) = *CCL5*, (**d**) = *IL1RN*, (**e**) = *CCL21*. The x-axis shows survival time in days; the y-axis shows survival probability. ns = nonsignificant. n = number of specimen analyzed for gene expression or number of patients in the survival curve computation.

**Figure 4:**
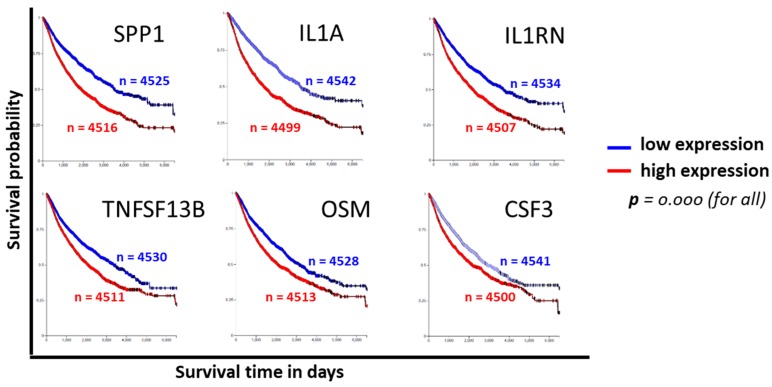
Cytokine genes identified as CPT-responsive were probed for clinical significance in the pan-cancer (PANCAN) TCGA database using the UCSD XenaBrowser platform. Kaplan–Meier patient survival curves for the top six genes with a highly significant (*p* = 0.000) survival probability based on the level of gene expression are shown. n = number of patients in the survival curve computation.

To evaluate the clinical relevance of cytokines induced by exposure to CPT, publicly available functional genomics databases were probed using UALCAN and XenaBrowser analysis platforms to correlate the expression of genes most modulated by CPT. We first examined the gene expressions and the patient survival probability for the 22 upregulated cytokines in the COAD dataset through the UALCAN portal. A gene expression comparison of profiles between normal tissues and primary tumors showed that SPP1 and IL1RN levels were higher in tumors, and that IL-16, CCL21, and CCL5 levels were lower (Figure 3a–e). Only the expression of SPP1 had a correlation with patient survival, for which a low expression of the cytokine was associated with better survival (*p* = 0.02). Next, we examined the significance of CPT-responsive genes in a larger database (PANCAN) through the UCSD XenaBrowser portal, covering pan-cancer datasets from more than 4000 patients. Kaplan–Meier plots were analyzed for each of the CPT-responsive genes. Plots from this profiling (Figure 4) revealed that six of the cytokines (SPP1, IL-1A, IL1RN, TNFSF13B, OSM, and CSF3) had associations between low expression and higher patient survival probability (*p* = 0.000). Since IL-1A, TNFSF13B, OSM, and CSF3 did not show an association with patient survival in the COAD dataset, we presume that these cytokines are more relevant in the biology or therapy of cancer types other than colon cancer. Expression profiles and survival probability curves for the remaining CPT-responsive cytokine genes, including CD274 (PD-L1), are shown as Supplementary Figures S1 and S2.

## 4. Discussion

In this report, we show that the chemotherapeutic drug CPT induces the upregulation of PD-L1 and other cytokines that modulate the attraction, migration, and functions of immune cells, primarily T-cells. Among more than 84 cytokines interrogated, 26 were modulated by the treatment of the cells with CPT. About 40% of these were regulators of T-cell functions, suggesting that the tumor cell response to treatment with conventional chemotherapy may create a microenvironment that attracts and regulates immune cells. 

Currently approved checkpoint inhibition therapies are directed toward blocking the PD-1/PD-L1 and CTLA4-CD28 interactions that regulate T-cell functions [26]. Hence, we determined if CPT modulates the expression of PD-L1. Functional regulation between SPP1 and PD-L1 has recently been reported [27]. Results obtained from immunoblotting, ELISA, and flow cytometry assays indicated that the expression of PD-L1 is upregulated by CPT. The response of PD-L1 did not positively correlate with the concentration of CPT used to treat these cells, as the lowest concentration of CPT induced the highest expression of the protein. The implications of this negative correlation should be investigated. We note that the potential for improved efficacy would be high for a low-dose CPT regimen (such as metronomic therapy [28,29,30,31]) combined with immunotherapy for checkpoint inhibition. Our current findings suggest that the sequential treatment of advanced CRC with a metronomic CPT regimen followed by checkpoint inhibition immunotherapy may be a good starting point. Chemotherapy of advanced CRC presently utilizes regimens that include 5-FU, oxaliplatin, or irinothecan/camptothecin as a component of standard care. Cells treated with 5-FU show an increase in the expression of PD-L1 [3], and cisplatin-resistant lung cancer cells express higher levels of PD-L1 [4], suggesting that upregulation of immunoregulatory mechanisms may be a common feedback mechanism of cancer cells exposed to DNA-damaging drugs or other therapies [32,33]. Although we did not extensively test 5-FU or oxaliplatin, the findings that colon cancer therapy agents induce checkpoint regulatory proteins need further study to identify the conditions under which chemoimmunotherapy combinations yield the best efficacy with the fewest side effects. Therefore, preclinical and clinical testing of these combinations is needed. 

Examination of the clinical profiles of these genes in publicly available databases showed that expressions of some cytokines (SPP1, IL1A, IL1RN, OSM, CSF3, and TNFSF13B) were increased in primary tumors compared to normal tissues, suggesting interactions among immune cells, tumor cells, and other cells through cytokines during tumor development. 

## 5. Conclusions

Colon cancer cells react to chemotherapy by upregulating or downregulating immunoregulatory molecules. Our findings that PD-L1 is upregulated and that additional cytokines may be elevated or suppressed upon chemotherapy provides an opportunity to determine whether combination strategies are needed to block or enhance the cytokine milieu. A mechanistic understanding of the functions of the most pertinent cytokines and their interactions with chemotherapy and radiation are of importance to improve therapeutic outcomes. 

## Figures and Tables

**Table 1:** Functional annotation of cytokines upregulated by treatment of SW620 cells with CPT. The list was sorted in decreasing order for fold expression induced by 1 µM CPT. Summaries of primary functions were collected from Gene Cards annotations (www.genecards.org).

Gene	Overall Upregulated by CPT Treatment (Fold Change vs. Control)		
	1 µM	5 µM	Major Functions Summary
SPP1	47.2	32.6	Secretes Phospho-Protein; osteoclast attachment; enhances IL-12 and reduces IL-10 production; induces PD-L1, promotes tumor [19]
IL-12B	45.1	104.3	Interleukin 12B; growth factor for activated T and NK-cells
CCL22	36.9	20	C-C Motif Chemokine Ligand 22; chemotactic for activated T cells, monocytes, DC, and NK cells; attracts T-regs [20]
IL-24	34.4	42.4	Interleukin 24; terminal differentiation of melanoma cell, may be pro-apoptotic; reduces tumor cell migration [21]
CXCL10	29.1	15	C-X-C Motif Chemokine Ligand 10; attracts and stimulates T cells, monocytes and NK cells; antimicrobial; anti-angiogenic [22]
IL1RN	27.9	27.4	Interleukin 1 Receptor Antagonist; inhibits IL-1a and b binding to IL1R, antagonist for IL-1; anti-inflammatory
TNF	26.9	75.3	Tumor Necrosis Factor; inflammatory signaling, induces IL-12, impairs T-reg function
CSF3	24.7	15.1	Colony Stimulating Factor 3; production, differentiation, and function of granulocytes
LTA	16.3	82.4	Lymphotoxin Alpha; inflammatory, immunostimulatory, antiviral; cytotoxic to tumor cells, binds TNF receptor (TNFR)
IL-17F	12.7	74.5	Interleukin 17F; stimulates peripheral blood mononuclear cells (PBMC), T-cells, and the production of IL-6, IL-8, CSF2, potentially anti-angiogenic; protumorigenic [23]
CCL2	10.9	2.9	C-C Motif Chemokine Ligand 2; tactic for monocytes and basophils, enhances monocyte anti-tumor activity
CCL17	10.7	16.3	C-C Motif Chemokine Ligand 17; tactic for T-cells, antimicrobial
CCL5	5.3	13.7	C-C Motif Chemokine Ligand 5; tactic for monocytes, Th cells and eosinophils; suppresses HIV replication; aka RANTES
IL-22	5.2	35.5	Interleukin 22; in vivo inflammatory reactions; protumorigenic in established cancer [24]
IL-5	4.3	24.6	Interleukin 5; growth and differentiation of B cells and eosinophils; clusters on Chromosome 5 with IL-4, IL-13 and CSF2 (all anti-inflammatory); eosinophil chemotaxis [25]
IL-13	3.6	10.5	Interleukin 13; regulation of B cell differentiation, anti-inflammatory, produced by Th2 cells, together with IL-10, IL-4, and IL-5
IL-16	2	4.6	Interleukin 16; stimulate migration of CD4 T cells, monocytes and eosinophils; primes CD4 cells for IL-2 and IL-15
CCL21	1.8	8.5	C-C Motif Chemokine Ligand 21; tactic for T-cells; homing of lymphocytes to secondary lymphoid organs
ADIPOQ	1.6	4.8	Adiponectin, C1Q And Collagen Domain Containing; anti-inflammatory adipokine, controls fat metabolism and insulin sensitivity, exclusive to adipose tissue
IL-1A	1.6	3.6	Interleukin 1A; inflammatory; thymocyte proliferation, B-cell maturation; secreted by Th1 and innate immune cells, with interferon (IFNγ), IL-6, tumor necrosis factor (TNFα)
OSM	1.2	5.4	Oncostatin-M; growth regulator, inhibits tumor cells; regulates IL-6, GM-CSF, and G-CSF production from endothelial cells
TNFSF13B	−1.4	3.2	TNF superfamily 13B; Potent B-cell activator

## Supplementary Materials

The following are available online at https://www.mdpi.com/2305-6320/6/2/51/s1, Figure S1: Expression profiles and patient survival probabilities for additional CPT-responsive cytokine genes, Figure S2: Expression profile heat map for 22 CPT-responsive cytokine genes identified using the TCGA COAD dataset, Table S1: Cytokines with overall downregulation by the treatment of SW620 cells with CPT.
