# Supplementary material for: Camptothecin Induces PD-L1 and Immunomodulatory Cytokines in Colon Cancer Cells
Source: Medicines (Basel). 2019 Apr 24;6(2):51. doi: 10.3390/medicines6020051 (PMC6631458; doi:10.3390/medicines6020051)
Supplement: Supplementary file 1 [file medicines-06-00051-s001.pdf]

Supplementary Materials: Camptothecin Induces PD-L1 and Immunomodulatory Cytokines in Colon Cancer Cells

Deepa Bedi, Henry J. Henderson, Upender Manne and Temesgen Samuel

Table S1. Cytokines with an overall downregulation by treatment of SW620 cells with CPT. The list was sorted in decreasing order for fold expression reduced by 1 μM CPT.

**Gene**	**Overall Down by CPT Treatment vs Control**		
	1 μM	5 μM	Major functions summary
XCL1	-10.9	-13.4	X-C Motif Chemokine Ligand 1; Chemotactic for T-cells; antimicrobial
C5	-4.6	-5.1	Complement component 5
IL-27	-2.9	-4.8	Interleukin 27; Potentiate TH1 response; suppress pro-inflammatory cytokines IL2, IL4, IL5, IL6; Inhibit TH17 activity and IL17 production
VEGFA	-2.0	-3.4	Vascular Endothelial Growth Factor A; Endothelial cell mitogen; Induces endothelial cell migration, and angiogenesis; Increase permeability
FASLG	-1.9	-3.0	Fas Ligand; T-cell apoptosis
HPRT1	-1.5	-2.4	Hypoxanthine Phosphoribosyltransferase 1; Purine nucleotide synthesis

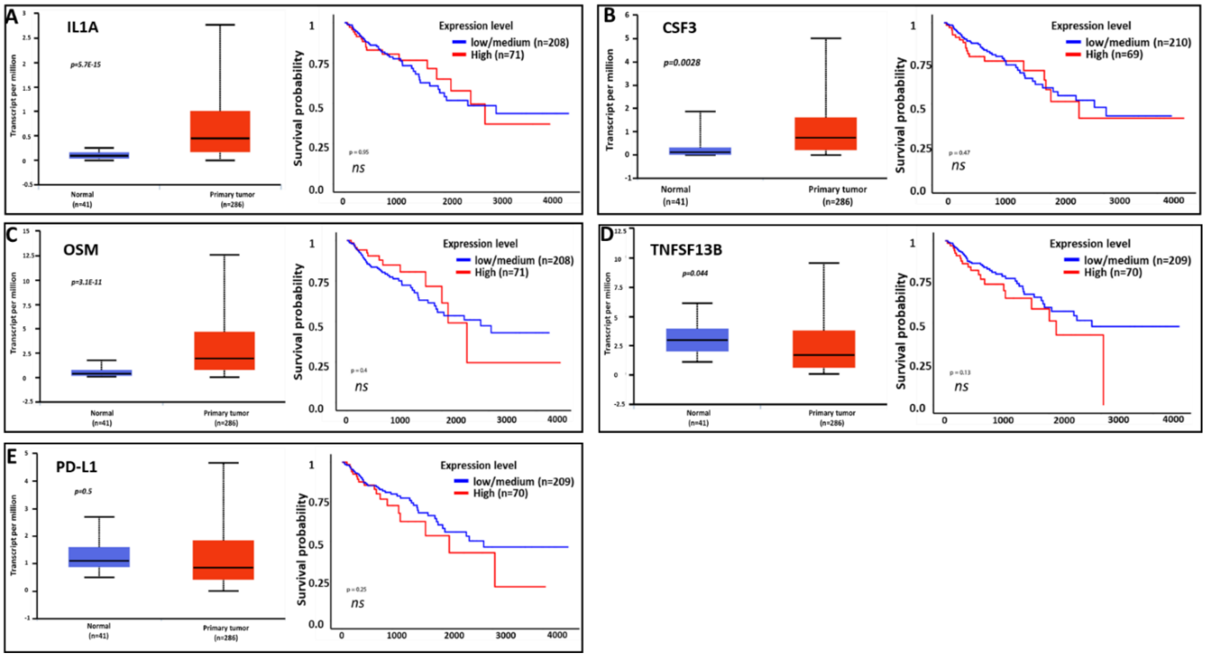

Figure S1. Expression profiles and patient survival probabilities for additional CPT-responsive cytokine genes. The TCGA database was probed for clinical significance by using the UALCAN portal. Graphs (**A**–**E**) for 5 additional genes, IL-1A, CSF3, OSM, TNFSF13B, and PD-L1, respectively, with their corresponding significance values in expression and/or survival probability are shown. Bar graphs show comparison of gene expression between normal and primary tumor, whereas the line graphs show effect of gene expression level on COAD patient survival. *n* is the sample size. *ns* = not significant. Note that, in the COAD dataset, PD-L1 expression is not significantly different between normal and primary tumors, nor are its expression levels associated with patient survival.

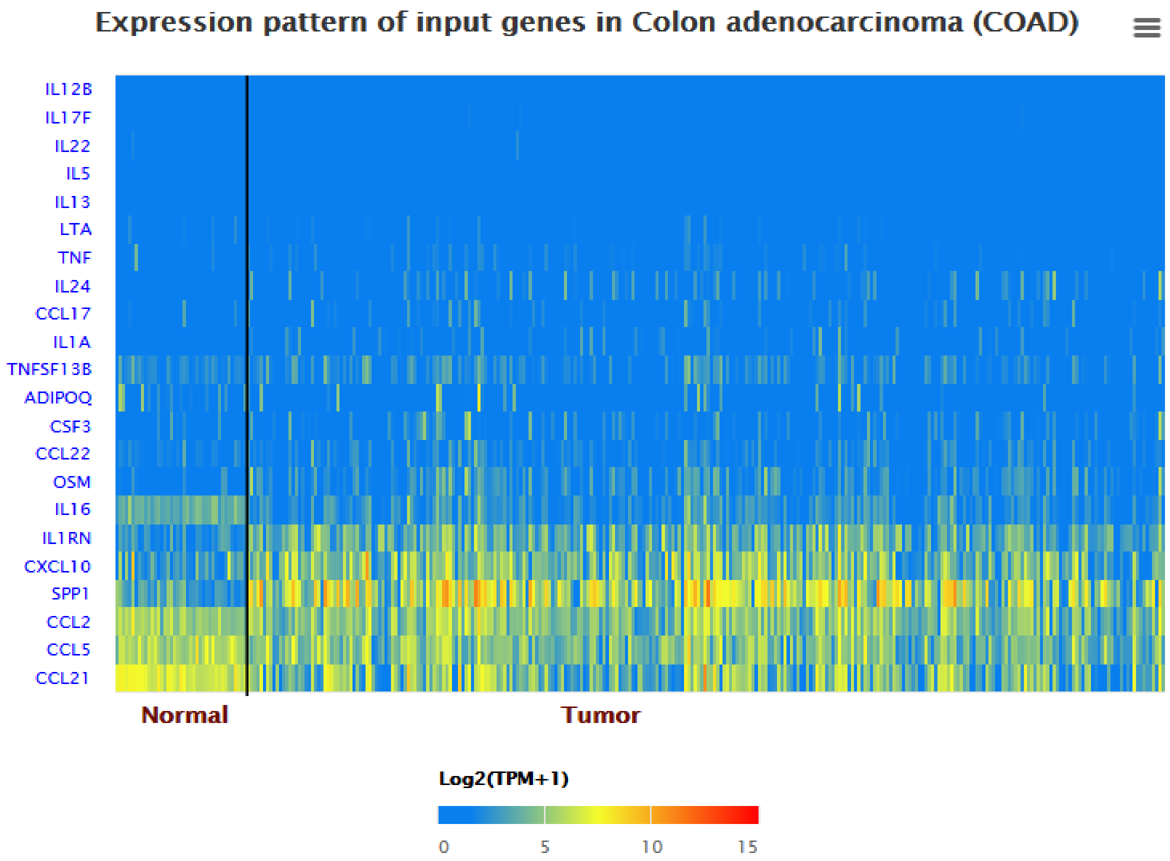

Figure S2. Expression profile heat map for 22 CPT-responsive cytokine genes identified using the TCGA COAD dataset showing differential expression between normal and tumor tissues of a number of CPT-responsive genes.
